# *COL4A1* gene mutations and perinatal intracranial hemorrhage in neonates: case reports and literature review

**DOI:** 10.3389/fped.2024.1417873

**Published:** 2024-06-18

**Authors:** Iliana Bersani, Sara Ronci, Immacolata Savarese, Fiammetta Piersigilli, Alessia Micalizzi, Chiara Maddaloni, Andrea Dotta, Annabella Braguglia, Daniela Longo, Francesca Campi

**Affiliations:** ^1^ Neonatal Intensive and Sub-Intensive Care Unit, Department of Medical and Surgical Neonatology, Bambino Gesù Children’s Hospital, IRCCS, Rome, Italy; ^2^ Neonatal Intensive Care Unit, Department of Pediatrics, Cliniques Universitaires Saint Luc, Université Catholique de Louvain, Bruxelles, Belgium; ^3^ Translational Cytogenomics Research Unit, Bambino Gesù Children’s Hospital, IRCCS, Rome, Italy; ^4^ Neuroradiology Unit, Imaging Department, Bambino Gesù Children’s Hospital, IRCCS, Rome, Italy

**Keywords:** collagen type 4, *COL4A1* gene, neonate, intracerebral hemorrhage, porencephaly

## Abstract

Intracranial hemorrhage may represent a complication of the perinatal period that affects neonatal morbidity and mortality. Very poor data exist about a possible association between mutations of the type IV collagen a1 chain (*COL4A1*) gene and the development of intracranial hemorrhage, and only sporadic reports focus on intracerebral bleedings already developing *in utero* or in the neonatal period in infants with such a mutation. This study presents a case series of term neonates affected by intracranial hemorrhage, with no apparent risk factors for the development of this condition, who were carriers of *COL4A1* gene variants. This study also provides a review of the most recent scientific literature on this topic, specifically focusing on the available scientific data dealing with the perinatal period.

## Introduction

Intracranial hemorrhage (ICH) develops most often in premature newborns, while its occurrence is relatively uncommon and may represent an incidental finding in term neonates (1, 2). Thanks to increased clinical awareness and improved neuroimaging techniques, however, ICH at term has been diagnosed more often in recent years (1, 3). In some cases, the clinical impact of such a condition may deeply influence the perinatal course and affect neonatal morbidity and mortality (4–7). ICH may develop *in utero* or after birth. The rates of fetal ICH are 0.46 per 1,000 deliveries and 0.9 per 1,000 pregnancies at referral centers (8). In full-term infants, possible risk factors for the development of perinatal cerebral bleeding include infections, malformations, asphyxia, and thrombophilia (4, 5, 7, 9). Besides these conditions, familial susceptibility with a genetic predisposition to a hereditary form of porencephaly may play a role, but available data are still inconsistent.

Type IV collagen a1 chain (*COL4A1*) gene mutations are responsible for a hereditary autosomal dominant cerebrovascular disease, characterized by a wide phenotypical spectrum, which may include the development of ICH. To date, only a relatively small number of neonates carrying *COL4A1* gene mutations and developing ICHs has been reported (10–15). However, the diagnosis of *COL4A1* gene mutations is probably underestimated because of the failure to search for the mutation because of its heterogeneous clinical presentation. When focusing on the perinatal period, the data available are even more infrequent, and the exact clinical features of such disorders are still far from clear.

The aim of this study was to describe the case of two term neonates affected by ICH of apparently unclear origin who showed mutations of the *COL4A1* gene, expanding the spectrum of perinatal disease attributable to *COL4A1* mutations and adding information in a field where knowledge is still far from clear. This study also provides a review of the currently available scientific literature on this topic, specifically focusing on the perinatal period.

## Case reports

We describe the clinical history of two neonates with apparently unexplainable ICH. A *COL4A1* gene mutation was detected in both patients.

### Patient 1

**Figure 1:**
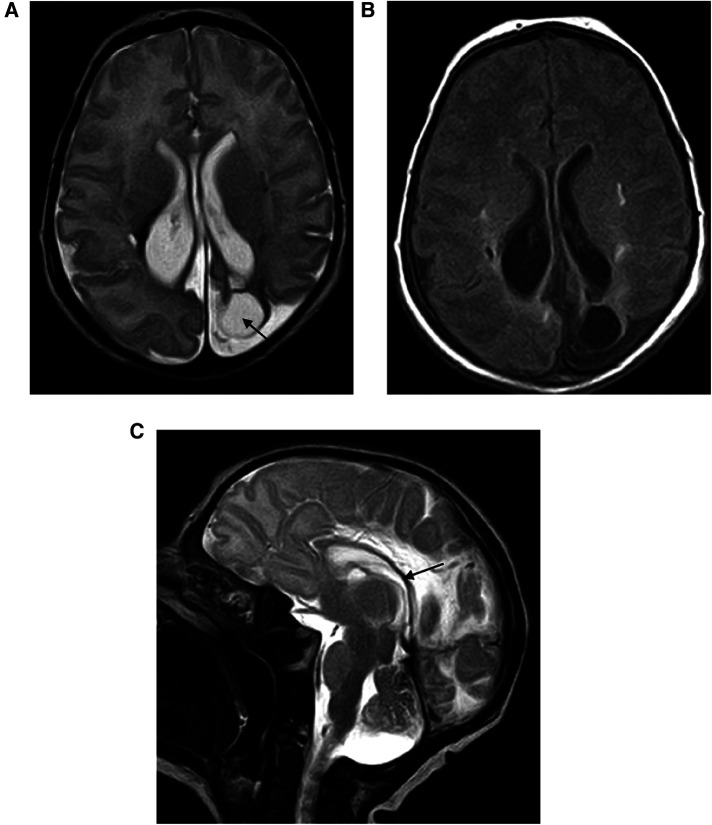
(**A**,**B**) Encephalomalacia secondary to parenchymal hemorrhage: axial T2-weighted image shows the limited area of parietal encephalomalacia (black arrow) with *ex vacuo* enlargement of the lateral ventricle. (**C**) Corpus callosum thickness reduction (black arrow).

Patient 1 (Table 1) was a male neonate born at a gestational age (GA) of 40 weeks by spontaneous delivery, with a birth weight (BW) of 2,900 g. One month before delivery, microcephaly and polihydramnios were detected. Amniocentesis was normal and maternal TORCH assessment was negative for recent infections. At birth, the neonate showed normal cardiorespiratory adaptation (Apgar score at 1 min: 9; 5 min: 10). Cerebral ultrasound (CUS) could not be performed because of the reduced diameter of the front cranial fontanel. Electroencephalography (EEG), performed on day 6 of life, was normal. Magnetic resonance imaging (MRI) of the brain, performed on day 9 of life, showed a volumetric reduction of the brain with a simplification of cortical convolutions, polycystic glio-malacic lesions located in the left parietal and occipital cortical-subcortical region and in the right parietal-mesial region, hemosiderin deposits suggestive of previous hemorrhage in the right posterior paraventricular region, asymmetry of the posterior horns of the lateral ventricles (right > left), and reduced thickness of the intermediate segment and of the splenium of the corpus callosum. Diffusion-weighted imaging (DWI) did not highlight any lesion suggestive of recent ischemic damage (Figure 1). On the day 17 of life, the neonate developed an episode of diffuse muscular hypertonus and gaze deviation. The EEG performed on this occasion showed right focal specific lesions and therapy with barbiturate was therefore begun. The subsequent EEG was normal.

**Table 1:** Neonatal clinical features.

	Patient 1	Patient 2
Gestational age (weeks)	40	38^+1^
Birth weight (g)	2,900	3,870
Sex	Male	Male
Delivery mode	Spontaneous	Spontaneous
Apgar score (1'–5')	9–10	9–10
Creatinine (mg/dl)	0.33	0.35
Platelets (10^3 ^/μl)	604	254
Prothrombin time (s)	11.9	13.3
Partial thromboplastin time (s)	31	33.5
INR	0.91	1.11
Protein C (%)	47	40
Protein S (%)	69 (slightly increased)	71 (slightly increased)
APC resistance (s)	128	125.8
Homocysteinemia (μmol/L)	9.8	5.22
Prothrombin gene mutation	Negative	Negative
Factor V gene mutation	Negative	Negative
*MTHFR* gene mutation	Heterozygosity	Heterozygosity
Cardiac assessment	*Foramen ovale*	Bovine aortic arch, *foramen ovale*
Ocular assessment	Normal	Normal
Audiological assessment	Normal	Normal
Urinary assessment	Normal	Monolateral pyelectasia + megaurether

INR, international normalized ratio.

After the first month of life, the infant developed muscular hypertonus of the four limbs, axial hypotonia, reduced reactivity, incomplete Moro reflex, right head deviation, and dysphagia.

The infant’s audiological and ocular assessments were normal. Renal function was normal. Thrombophilia testing was performed; prothrombin time, partial thromboplastin time, bleeding time, fibrinogen, antithrombin III protein C, protein S, activated protein C (APC) resistance, homocysteinemia, and testing for mutations in the prothrombin and factor V were all normal. A heterozygosity of the methyltetrahydrofolate reductase (*MTHFR*) gene (C677T) was detected. Immunoglobulins for *rubeola*, *herpes* viruses 1/2, *Toxoplasma gondii*, *cytomegalovirus*, and urinary polymerase chain reaction for *cytomegalovirus* excluded viral infections.

After the proband's parents provided written informed consent, molecular analyses of *COL4A1* were performed using a custom panel [NimbleGen SeqCap Exome Enrichment Kit (Roche Sequencing Solution, CA, USA)] according to the manufacturer's protocol and sequenced on the Illumina platform. Annotated data were filtered out to exclude intronic variants, synonymous variants not predicted to affect splice sites, and variants with minor allele frequency (MAF) of ≥0.1% in the Genome Aggregation Database (gnomAD). Trio analysis identified the heterozygous missense variant c.2705C>G (p.Pro902Arg) of the *COL4A1* gene (NM_001845) in maternal segregation in our patient.

The *COL4A1* gene is an autosomal dominant gene of susceptibility to cerebral hemorrhages. The variant was not known in the scientific literature and was reported in the reference population database (gnomAD AF: 0.0004351; dbSNP: rs146134172; ClinVar ID: 80023). At the comparative genomic hybridization (CGH) array, the infant was also positive for the microduplication 16q24.3 with paternal segregation.

The copy number variants (CNVs) of region 16q24.3 are frequently associated with KBG syndrome. KBG syndrome is often caused by mutations in the *AKNRD11* gene, microdeletions of 16q24.3, and, less frequently, by microduplications of this same region (16). Furthermore, the patient did not have phenotypic features peculiar to KBG syndrome, such as triangular face, hypertelorism, brachycephaly, synophyrys, prominent nasal bridge, and elongated philtrum. Furthermore, he had never experienced a single seizure episode that then resolved. Therefore, this diagnosis was excluded. As integration, the analysis of genes associated with microcephaly was performed and was negative.

The patient received rehabilitative physiotherapy during hospitalization and after discharge as well as speech therapy support. Percutaneous endoscopic gastrostomy and jejunostomy were performed because of persistent dysphagia, feeding difficulties, and gastroesophageal reflux. A follow-up at 7 years of age showed that the infant developed severe neurodevelopmental delay, spastic tetraparesis, and relational and language disorders.

### Patient 2

**Figure 2:**
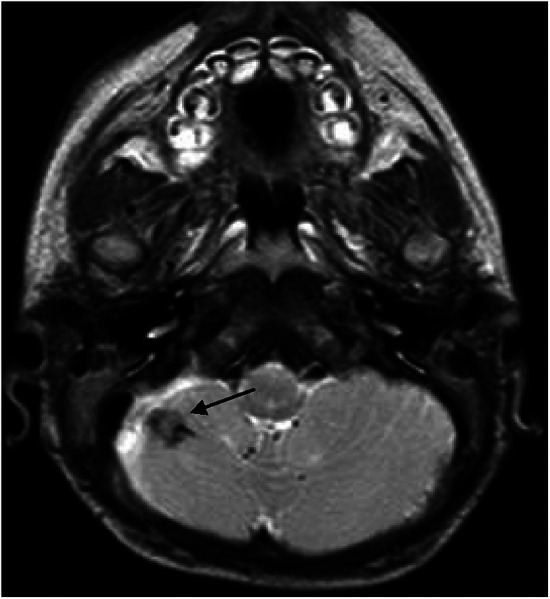
Small parenchymal hemorrhage in the right cerebellar hemisphere (black arrows).

Patient 2 (Table 1) was a male neonate born at a GA of 38 weeks by spontaneous delivery, with a BW of 3,870 g and Apgar scores of 9 and 10 at 1 and 5 min, respectively. Pregnancy was uneventful except for placental dysfunction during the first trimester, which was treated with aspirin. The early postnatal period was normal. On day 7 of life, the neonate developed jaundice, multiple vomiting episodes, and irritability. CUS showed asymmetric dilatation of the lateral ventricles with moderate (grade II–III) bilateral intraventricular hemorrhage (IVH) and dilatation of the third ventricle. Brain MRI confirmed these findings and highlighted further hemorrhage in the context of the posterior cranial fossa and small parenchymal hemorrhage in the right cerebellar hemisphere, in the cisterns of the skull base, and in the left hemispheric extra-axial site (Figure 2). EEG showed electric seizures on the left occipital hemisphere, which were treated with barbiturate.

As a result of worsening dilatation of the cerebral ventricles, a ventricular drain connected to a subcutaneous reservoir was inserted 1 week later, which was then replaced with a ventriculo-peritoneal shunt. The patient’s ocular and audiological assessments were normal. Left pyelectasia and megaureter were present and were subsequently treated with endoscopic dilation of the left uretero-vesical joint and ureteral stent placement. Extensive laboratory investigations, including complete blood count, renal function, C-reactive protein, prothrombin time, partial thromboplastin time, protein C, protein S, APC resistance, bleeding time, homocysteinemia, and testing for mutations in the prothrombin and factor V genes were all normal. The heterozygosity of the *MTHFR* gene (C677T) was highlighted. At the follow-up carried out at 3 years of age, the patient’s neurodevelopment was normal.

After the proband's parents provided written informed consent, a molecular analysis of *COL4A1* was performed using the custom panel with the same technique as described in the first case. Trio analysis identified heterozygous missense variant c.413C>G (p.Pro138Arg) of the *COL4A1* gene (NM_001845) present in the newborn and in his mother. The variant was neither known in the scientific literature nor reported in the reference population database (gnomAD, HGMD). *In silico* bioinformatic prediction tools indicated that the variant was pathogenic.

### Literature review

To review the literature about *COL4A1* and perinatal intracranial hemorrhage in neonates, an extensive literature search in the MEDLINE database (via PubMed) was performed up to the year 2000. The keywords “COL4A1” AND “perinatal intracranial hemorrhage” OR “neonatal intracranial hemorrhage” were also searched as entry terms. All retrieved articles were screened, and then full texts of records deemed eligible for inclusion were assessed. References in the relevant papers were also reviewed and further articles were added if necessary. Papers written in languages other than English were excluded.

In Table 2, we systemically collected and summarized information on patients’ characteristics, age at diagnosis, and genetic diagnosis, and we compared them to our case.

**Table 2:** Perinatal intracerebral hemorrhage associated with *COL4A1* gene mutations.

Reference	GA (weeks)	Delivery mode	Cardiorespiratory adaptation at birth	ICH onset	Associated anomalies	*COL4A1* gene mutations	Inherited/*de novo*
De Vries et al. (17)	33^+5^	Spontaneous	Normal	Prenatal	Strabismus, quadrant hemianopia	Both patients (siblings): heterozygous G C change at nucleotide 4738 (c.G4738C) of exon 50, causing a glycine-to-arginine substitution at position 1580 of the procollagen-4 alfa-1 chain (p.G1580R)	Inherited
	31	Spontaneous	Apgar 1': 4, 5': 8	Prenatal	Strabismus	Both patients (dizygotic twins): c.4582–4586dupCCCATG insertion in exon 4 causing a third proline-methionine repeat inserted into the highly conserved noncollagenous (NC1) domain	Inherited
Bilguvar et al. (18)	24	Spontaneous	Apgar 1': 1, 5': 1	Postnatal	—		Inherited
	24	Spontaneous	Apgar 1': 2, 5': 5	Postnatal	—	Heterozygous c.2245G>A mutation, leading to glycine-to-serine substitution at protein level (p.Gly749Ser)	Inherited
Vermeulen et al. (19)	31^+1^	NA	NA	Prenatal	Microcephaly, IUGR		Inherited
	40	NA	NA	Prenatal	Microcephaly, IUGR	Heterozygous c.4150G>A mutation, leading to glycine-to-serine substitution at protein level (p.Gly1384Ser).	*De novo*
Meuwissen et al. (20)	37	NA	NA	Prenatal	Right kidney, renal artery, femoral vein agenesis, left-sided renal cystic lesions	Heterozygous c.2545G T mutation (p.G808V) in exon 31	*De novo*
	40	NA	NA	Prenatal	Optic atrophy	c.2716 1G A splice site mutation in exon 33	*De novo*
	40	NA	NA	Prenatal	Bilateral small corneas and cataracts	c.3022G A mutation (p.G1008R) in exon 36	Inherited (mosaic)
	37	NA	NA	Prenatal	IUGR, microcephaly, bilateral cataract	c.3130G C mutation (p.G1044R) in exon 37	*De novo*
Lichtenbelt et al. (21)	41^+6^	Spontaneous	Normal	Prenatal	Retinal hemorrhages	Pathogenic missense mutation (G1103R)	*De novo*
Takenouchi et al. (22)	35^+6^	Cesarean delivery	Normal	Prenatal	Hemolytic anemia	Heterozygous c.3715G>A missense mutation in exon 42 of *COL4A1* (NM_001845), leading to glycine-to-arginine substitution at the triple helical region of *COL4A1* (p.Gly1239Arg)	Inherited
Colin et al. (23)	Pregnancy termination (34)	—	—	Prenatal	Bilateral cataract	Heterozygous novel missense mutation c.2317G4A (p.Gly773Arg)	Inherited
	Pregnancy termination (34)	—	—	Prenatal	Bilateral Cataract	Heterozygous novel missense mutation c.3005G4A (p.Gly1002Asp)	Unknown
Meuwissen et al. (24)	1/11: 25 10/11: >30	NA	NA	Prenatal	Cataract	c.3280G>C,p.Gly1094Arg	Inherited
		NA	NA	Prenatal	Microcornea, optic atrophy, hypermetropia, pyelectasia	c.4150+1G>A	*De novo*
		NA	NA	Prenatal	Microcephaly	c.4739G>C, p.Gly1580Ala	*De novo*
		NA	NA	Prenatal	Microcephaly, hypermetropia, mild hydronephrosis	c.1964G>A, p.Gly655Glu	Inherited
		NA	NA	Prenatal	Sporadic fetal movements, hydranencephaly	c.2636G>A, p.Gly879Glu	*De novo*
		NA	NA	Postnatal	retinal arterial tortuosity, autism	c.2581G>A, p.Gly861Ser	*De novo*
		NA	NA	Postnatal	Hypermetrophya	c.2581G>A, p.Gly861Ser	Inherited
		NA	NA	Postnatal	Supraventricular arrhythmia	c.1870G>T, p.G624*	Inherited
		NA	NA	Postnatal	Unknown	c.1870G>T,p.G624*	Unknown
		NA	NA	Postnatal	—	c.2008G>A, p.Gly670Arg	*De novo*
		NA	NA	Postnatal	Hypermetropia, visual field defect, myopathy, muscle cramps	c.3770G>A, p.Gly1257Glu	Inherited?
Durrani-Kolarik et al. (25)	39	Cesarean delivery	Normal	Prenatal	IUGR, proptosis, minimal visibility of the sclera, cloudy cornea, abnormal size + shape of pupil and iris	Point mutation at Gly785Glu	*De novo*
Grego et al. (26)	38	Spontaneous	Normal	Prenatal	IUGR, optic nerve hypoplasia, bilateral cataract	Heterozygous mutation c.2716+2T>C, affecting a consensus sequence for splicing	*De novo*
Maurice et al. (27)	Pregnancy termination (26)	—	—	Prenatal	Polyvisceral hemorrhagic suffusion	*COL4A1*: c.563G>A, p.(G188E) (ACMG class 5)	Inherited
	Pregnancy termination (24)	—	—	Prenatal	—	*COL4A1*: c.4756C>A, p.(H1586N)(ACMGclass4)	*De novo*
	Pregnancy termination (22)	—	—	Prenatal	—	*COL4A1*: c.1946G>A, p.(G655E)(ACMG class 5) (Karyotype 47,XXX)	*De novo*
	Pregnancy termination (22)	—	—	Prenatal	—	*COL4A1*: c.4150+1G>A (ACMG class 4)	*De novo*
	Pregnancy termination (32)	—	—	Prenatal	—	*COL4A1*: c.2008G>A, p.(G670R) (ACMG class 5)	*De novo*
	Pregnancy termination (22)	—	—	Prenatal	—	*COL4A1*: c.2642T>C, p.(M881T)	Unknown

IUGR, intrauterine growth retardation; NA, not available.

A total of 33 cases were reported in the literature before our case. In 25 of the 33 cases, the onset of ICH was prenatal, and pregnancy was terminated in 8 cases.

## Discussion

*COL4A1* and type IV collagen a2 chain (*COL4A2*) form the (a1)2(a2) IV heterotrimers and represent key components of basement membranes (28–31). *COL4A1* is detectable ubiquitously in all tissues, including the vasculature, renal glomeruli, ocular structures, and muscles, and plays a role in the development of basement membrane during embryogenesis, in the cohesiveness of these membranes, and in the maintenance of vascular tone (32–34). The *COL4A1* gene is located on the telomeric region of 13q (13q34) and is made up of 52 exons (35). Mutations of the *COL4A1* gene may lead to a wide range of abnormalities variably affecting the brain (including the development of ICHs), retinal vasculature, ocular structures, renal glomeruli, and muscles, but no clear genotype–phenotype association has been identified yet, even within the same family (15). *COL4A1* mutations are inherited as an autosomal dominant trait and have near 100% penetrance with variable expression; *de novo* mutations or low-level parental mosaicism have been described (25).

Neonates described in the present case series were both born at term after pregnancies with no history of infections, traumatic injuries, or thrombotic events and had a normal cardiorespiratory adaptation at birth. The heterozygosity of the *MTHFR* gene was highlighted in both cases, but no other anomalies were detected concerning the coagulative tests, platelet counts, and thrombophilia screening. ICH was diagnosed postnatally in both cases, although the radiologic findings of patient 1 showed the presence of glio-malacic lesions that were presumably of antenatal origin; even if it was not possible to assess the exact onset of the ventricular dilation detected in patient 2, the entity of the lesion was probably consistent with a prenatal origin. In our cases, prenatal MRI was not performed because of the late detection of anomalies during pregnancy in the first case and the low risk of pregnancy in the second case. However, as recently described by George et al. (36), prenatal *COL4A1*/*A2* variations can show a range of ICH affecting the frontal lobes and basal ganglia, such as minor and/or unifocal ICH, as well as multifocal and bilateral lesions. In all cases of prenatal ICH, genetic testing for *COL4A1*/*A2* variations should be taken into consideration because of the wide range of severity observed on fetal brain MRI. However, both patients came to our observation in the postnatal period; therefore, we had no other information regarding the decision-making process that took place during pregnancy.

The neurologic outcome was heterogeneous since it ranged from normal neurodevelopment (patient 2) to severely impaired neurologic development (patient 1).

To date, only a few authors have specifically investigated the role of *COL4A1* mutations in the pathophysiologic mechanisms leading to intracerebral bleeding, and most of them focused on pediatric/adult patients rather than on neonates (37–39). Vahedi et al. found that some *COL4A1* gene mutations may remain completely asymptomatic for years with no vascular changes on brain MRI, suggesting that the clinical expression may be heterogeneous even within the same family (37). Such findings are in agreement with subsequent data showing that brain MRI is abnormal in the majority of *COL4A1*-mutated patients (even in asymptomatic ones), but normal brain MRI may also be detectable in some mutation carriers with no brain manifestations (39).

Although there has been increasing interest regarding this condition in recent years, clinical experience about *COL4A1*-related perinatal ICHs only results from sporadic case reports (Table 1). Gould et al. were the first to demonstrate in both a mice model and in humans that mutations in the *COL4A1* gene may compromise the vascular basement membrane and lead to perinatal ICH (10). Since then, other authors reported the association between *COL4A1* gene mutation and perinatal ICHs, but no exact genotype–phenotype association has been identified yet.

The involvement of the central nervous system in the presence of *COL4A1* gene mutations varies widely from case to case in terms of onset timing, injury entity, and location (17–27, 40, 41). Features suggestive of *COL4A1* mutations include severe and/or multifocal hemorrhagic or ischemic-hemorrhagic cerebral lesions, especially when such injuries are associated with porencephaly (27).

A deeper understanding of the relationship between *COL4A1* mutations and the development of perinatal porencephaly may have important implications for disease prevention and optimization of perinatal care. Some authors suggested a possible interaction between environmental triggers and the genome (10). Although some neonatal cases were diagnosed after birth (as in our cases), the postnatal neuroimaging features were mostly consistent with an antenatal onset of ICH (Table 1).

It is crucial to emphasize the importance of the mother–placental–fetal triad in the developing of illness processes that cause brain abnormalities in fetuses or newborns. Gene–environment interactions affect the brain development of newborns, children, and the mother–placental–fetal triad in both the short and long term. These interactions start at conception (40). During the initial 1,000 days of brain maturation, critical/sensitive periods are more likely to result in lifelong developmental neuroplasticity (40). The hazardous stressor interaction between internal and external sources modifies the neural exposome by means of maladaptive developmental neuroplasticity. The first 1,000 days are when epilepsy and developmental problems mostly manifest (41).

Prenatal environmental factors, such as premature uterine contractions or other traumatic injuries, may activate some triggering mechanisms leading to vascular damage in at-risk patients (17, 42). It was hypothesized that specific preventive strategies, such as cesarean delivery instead of spontaneous delivery in at-risk individuals, might decrease the stress on the abnormal brain vasculature and, therefore, the severity of ICH in high-risk populations (10); however, further studies are needed. Nevertheless, according to the literature, perinatal ICHs occurred in neonates born both by spontaneous and cesarean delivery, and brain injury has sometimes already occurred during pregnancy (Table 2).

Finally, the neonates described in the literature were born either at term or prematurely (Table 2), and it might be put forward that some *COL4A1* mutations may carry a higher risk for preterm birth and/or higher susceptibility to environmental factors than others (17).

Regarding the importance of prevention, it is also important to highlight that many children with developmental disabilities live in low-resource countries low or middle income country (LMICs) or high-income desert countries (HICMDs).

In these places, it is even more important to implement strategies aimed at identifying at-risk pregnancies as early as possible to provide families with accurate diagnoses and subsequent effective interventions. To this end, a biosocial model was developed that emphasizes women's reproductive health with trimester-specific maternal and pediatric health interactions (43).

The ability to perform genetic, neurophysiological, and neuroimaging assessments improves clinical decision-making for more effective interventions before pathological expression.

Support for the family, especially in the case of neurological pathologies in which we know how fundamental the first 1,000 days of life are, continues in the postnatal period (40).

Synergy between obstetric and pediatric healthcare providers can reduce neurological morbidities (43).

In conclusion, mutations of the *COL4A1* gene as a potential risk factor for perinatal ICH are probably underestimated because of low clinical awareness, poor available data, and wide phenotypical spectrum; *COL4A1* mutations should be suspected when otherwise unexplainable ICHs occur prenatally or in the neonatal period; if *COL4A1* carriers are identified, familiar genetic counseling is advisable (37, 44); and genetic testing can be performed both prenatally and postnatally (24). There is still a lack of long-term follow-up data about the risk of ICH recurrence as long-term neurologic outcomes are missing. The current gap in knowledge in this field represents a call to action for clinicians/researchers and deserves further investigations.
